# Decreased catalytic function with altered sumoylation of DNA topoisomerase I in the nuclei of scleroderma fibroblasts

**DOI:** 10.1186/ar3435

**Published:** 2011-08-09

**Authors:** Xiaodong Zhou, Wei Lin, Filemon K Tan, Shervin Assassi, Mavin J Fritzler, Xinjian Guo, Roozbeh Sharif, Tom Xia, Syeling Lai, Frank C Arnett

**Affiliations:** 1 Division of Rheumatology, Department of Internal Medicine, University of Texas Health Science Center at Houston, Houston, TX 77030, USA; 2 Department of Medicine, University of Calgary, Calgary, AB T2N 1N4, Canada; 3 Rice University, Houston, Post Office Box 1892, TX 77030, USA; 4 Departmant of Pathology, Baylor College of Medicine, Houston, TX 77030, USA

## Abstract

**Introduction:**

Sumoylation is involved in nucleolus-nucleoplasm transport of DNA topoisomerase I (topo I), which may associate with changes of cellular and topo I functions. Skin fibroblasts of patients with systemic sclerosis (SSc) exhibit profibrotic cellular changes. The aims of this study were to examine the catalytic function and sumoylation of topo I in the nuclei of SSc fibroblasts, a major cell type involved in the fibrotic process.

**Methods:**

Eleven pairs of fibroblast strains obtained from nonlesional skin biopsies of SSc patients and age/sex/ethnicity-matched normal controls were examined for catalytic function of nuclear topo I. Immunoprecipitation (IP)-Western blots were used to examine sumoylation of fibroblast topo I. Real-time quantitative RT-PCR was used to measure transcript levels of SUMO1 and COL1A2 in the fibroblasts.

**Results:**

Topo I in nuclear extracts of SSc fibroblasts generally showed a significantly lower efficiency than that of normal fibroblasts in relaxing equivalent amounts of supercoiled DNA. Increased sumoylation of topo I was clearly observed in 7 of 11 SSc fibroblast strains. Inhibition of SUMO1 with SUMO1 siRNA improved the catalytic efficiency of topo I in the SSc fibroblasts. In contrast, sumoylation of recombinant topo I proteins reduced their catalytic function.

**Conclusions:**

The catalytic function of topo I was decreased in SSc fibroblasts, to which increased sumoylation of topo I may contribute.

## Introduction

Systemic sclerosis (SSc) is a human multi-system fibrotic disease with high morbidity and mortality but the etiology is largely unknown and the pathogenesis has yet to be clearly elucidated. Cutaneous fibrosis is a common clinical presentation and, based on the extent of skin involvement, SSc is classified into limited and diffuse cutaneous forms. The latter subset is characterized by more rapid progression of skin and visceral involvement, as well as poorer prognosis [1,2]. Skin fibroblasts obtained from SSc patients have been found to be profibrotic and to synthesize excessive amounts of ECM proteins, which contribute to tissue fibrosis [3]. It is believed that a possible defect in regulation of biological functions is present in SSc fibroblasts.

The majority of SSc patients (95%) have autoantibodies against various nuclear, nucleolar and cytoplasmic proteins, which include non-specific antinuclear antibodies (ANA) and a number of disease specific autoantibodies. Anti-DNA topoisomerase I (topo I) autoantibody is one of the disease-specific autoantibodies, and it occurs in 15 to 25% of patients [4-6]. A causal contribution of anti-topo I to the SSc phenotype is still unclear. There is no direct evidence indicating pathogenic roles of the antibodies. On the other hand, there is a strong association between anti-topo I autoantibody and the diffuse cutaneous form of SSc [5,6]. Levels of anti-topo I autoantibodies have been reported to correlate with disease severity and activity in SSc, and the lack of these antibodies conveys a better outcome in SSc [7]. In addition to anti-topo I, other SSc specific autoantibodies include those directed against centromeric proteins (ACA) that are associated with limited cutaneous disease, RNA polymerases (I, II and III) (ARA) and fibrillarin that are associated most often with diffuse skin involvement [8].

Topo I is a monomeric 100 kD nuclear protein that catalyzes the breaking and joining of DNA strands prior to transcription [9,10], and is associated with transcription, DNA replication and chromatin condensation. Topo I translocates between the nucleolus and the nucleoplasm, but is enriched in the nucleolus where there is a high level of transcription and replication of the ribosomal DNA [9,10]. Sumoylation is a post-translational modification, in which the substrates covalently attach the small ubiquitin-like modifier (SUMO) to lysine residues. Sumoylation is an important mechanism in regulating functions of target proteins and has been associated with the pathogenesis of autoimmune and inflammatory diseases, such as type I diabetes mellitus and rheumatoid arthritis [11,12]. Sumoylation of topo I was reported to facilitate its movement between the nucleolus and the nucleoplasm [13,14].

The goal of this study was to determine whether there is abnormal function, distribution and/or sumoylation of topo I in fibroblasts obtained from SSc patients that might associate with the presence of anti-nuclear and -nucleolar autoantibodies.

## Material and methods

### Dermal fibroblast cultures

Nonlesional skin biopsies (3 mm punch biopsies) were obtained from the upper arms of 11 SSc patients with disease of less than five years duration and 11 age- and gender-matched normal controls. All SSc patients fulfilled American College of Rheumatology criteria for SSc [15], and were positive for ANA. Two patients were positive for anti-topo I, four for ACA, two for ARA and one for anti-fibrillarin. Six patients had a diffused form of SSc, and five had limited SSc. Normal controls were undergoing dermatologic surgery and had no identified history of autoimmune diseases. All subjects provided informed consent and the study was approved by the Committee for the Protection of Human Subjects at The University of Texas Health Science Center at Houston.

Each skin sample was transported in Dulbecco's Modified Essential Media (DMEM) with 10% fetal calf serum (FCS) supplemented with penicillin and streptomycin for processing the same day. The tissue samples were washed in 70% ethanol, PBS and DMEM supplemented with 10% FCS. Cultured fibroblast cell strains were established by mincing tissues and placing them into 60 mm culture dishes secured by glass coverslips. The primary cultures were maintained in DMEM with 10% FCS and supplemented with penicillin and streptomycin. The early passage (< 5 passages) fibroblast strains were plated at a density of 2.5 × 10^5 ^cells in 35 mm plates and grown for assays accordingly.

### Catalytic function of topo I in SSc fibroblasts

Nuclear proteins were extracted from equal amounts of the cultured fibroblast cells by using nuclear extract kits (Active Motif, Carlsbad, CA, USA). The Topoisomerase I Assay kit (TopoGEN Inc., Port Orange, FL, USA) was used for measuring the catalytic function of topo I. Briefly, supercoiled DNA substrate (0.25 μg) (TopoGen, Inc.) was reacted with nuclear proteins containing topo I at serial dilutions. After 30-minute incubations at 37°C, the reaction was terminated with stop buffer (5% Sarkosyl, 0.125% bromophenol blue and 25% glycerol). The reaction mixtures were loaded and electrophoretically separated on a 1% agarose gel, and then stained with ethidium bromide. The catalytic activity of topo I was determined by measuring the intensity of the supercoiled DNA bands after reactions with a serial dilution of topo I in the nuclear extract of fibroblasts. A Bio-imaging system (Gene Genius, Syngene, Frederick, MD, USA) was used to scan the bands in agarose gel. The Gene Snap software (Syngene) was used to quantify the intensity of the bands. A total of 11 pairs of SSc and control fibroblast strains were examined with this assay.

#### Immunostaining

SSc and normal fibroblasts were grown in culture media as described above. After 7, 14 and 18 days, the cells were washed with PBS and fixed with 100% methanol at 4°C for two minutes. The cells were washed with PBS again, and incubated with serum from SSc patients (evenly pooled from four SSc patients) who had positive anti-topo I autoantibodies, or monoclonal antibodies of mouse anti-human topo I or mouse anti-human SUMO 1. This was followed by incubation with green fluorescent protein (GFP) tagged secondary antibodies (rabbit anti-human IgG antibodies and anti-mouse antibodies). Nuclei were visualized by counterstaining DNA with 4',6-diamidino-2-phenylindole (DAPI) (Vector Laboratory Inc., Burlingame, CA, USA). The images of fibroblasts with fluorescence labeled proteins were acquired using fluorescence microscopy (Nikon Eclipse TE2000-4., Melville, NY. USA).

### Western blotting

The protein concentration of nuclear extracts from cultured fibroblasts was measured using the standard curve in a TECAN spectrophotometer (Tecan Group Ltd., Switzerland, 8708 Mannedorf). Equal amounts of protein from each sample were subjected to SDS-polyacrylamide gel electrophoresis. Resolved proteins were transferred onto nitrocellulose membranes and incubated with 1:1,000 diluted primary antibodies including mouse anti-human topo I (ImmunoVision, Springdale, AR, USA), anti-human SUMO1 (ABGENT, San Diego, CA, USA) and anti-collagen type I, individually. The secondary antibody was a peroxidase-conjugated anti-mouse IgG (Amersham, Piscataway, NJ, USA). Specific proteins were detected by chemiluminescence using an Enhanced Chemiluminescence (ECL) system (Amersham). The intensity of the bands was quantified using ImageQuant software (Molecular Dynamics, Sunnyvale, CA, USA).

#### Immunoprecipitation (IP) Western blotting

Approximately 3.5 × 10^7 ^fibroblast cells of each subject were harvested by trypsinizing the adherent cells and washed twice with 25 ml ice-cold PBS containing phosphatase inhibitors. Cell pellets were then gently resuspended by 2 ml hypotonic buffer and nuclear extracts prepared and measured for protein concentration by a spectrophotometer as described above. Equal amounts of protein (500 ug) from each sample were subjected to immunoprecipitation (IP) with mouse anti-SUMO-1(GMP1, Invitrogen, Carlsbad, CA, USA) using nuclear complex co-IP kit (Active Motif, Carlsbad, CA), and then subjected to SDS-polyacrylamide gel electrophoresis. Resolved proteins were transferred onto nitrocellulose membranes and incubated with primary antibodies of mouse anti-human topo I (ImmunoVision) diluted to 1:1,000. The secondary antibody was a horseradish peroxidase-conjugated anti-mouse IgG (eBioscience, San Diego, CA, USA). Specific proteins were detected by chemiluminescence using Supersignal West Pico stable peroxide solution (Thermo Scientific, Rockford, IL, USA). The intensity of the bands was quantified using ImageQuant software (Molecular Dynamics).

### Inhibition of SUMO1 with siRNA transfection in fibroblasts

SUMO1 siRNAs were purchased from Invitrogen. Three SSc fibroblast strains that showed stronger sumoylation of topo I and weaker catalytic topo I function were used for transfection of SUMO1 siRNA. Briefly, the fibroblasts were grown at a density of 1.5 × 10^5 ^cells in 25-cm^2 ^flasks until confluency. The DMEM culture medium in each culture flask was replaced with Opti_MEM 1 (Invitrogen) without FCS. The fibroblasts were transfected with SUMO siRNA using Lipofectamine RNAiMAX (Invitrogen) at a concentration of 15 ug/ml. A fluorescein-labeled non-silencing control siRNA (Qiagen, Valencia, CA, USA) was used for detection of transfection efficiency. After 24 hours, the culture medium was replaced with normal DMEM. The fibroblasts were examined for gene and protein expression, as well as topo I catalytic function after 48- or 72-hour transfection.

### Sumoylation assay of topo I

A mixture containing recombinant topo I protein (TopoGEN Inc.), SUMO-1 protein (Active Motif), activating enzyme E1/conjugating enzyme E2 (Active Motif) and sumoylation buffer (15 mM ATP, 25 mM MgCl2 and 250 mM Tris-HCl) was incubated at 30°C for three hours. A mutant SUMO-1 protein (Active Motif) lacking sumoylation function was used as a negative control. The reaction was stopped with 5 mM EDTA and the recombinant human topo I with and without sumoylation were examined by Western blotting and topo I catalytic assays. The experiments were performed in triplicate.

### Quantitative reverse-transcriptase-polymerase chain reaction (RT-PCR) for measurement of SUMO1 expression, as well as COL1A2 expression after SUMO1 siRNA transfection

The primers and probes of SUMO1, COL1A2, 18S and GAPDH were obtained from Applied Biosystems (Assays-on-Demand product line; Foster City, CA, USA). Total RNA from each sample was extracted from the cultured fibroblasts described above using a total RNA kit from OMEGA Biotek (Norcross, GA, USA) after treatment with DNase I. Complementary DNA (cDNA) was synthesized using SuperScript II reverse transcriptase (Invitrogen). Synthesized cDNAs were mixed with primer/probe of SUMO1 or COL1A2 in 2 × TaqMan universal PCR buffer and then assayed on an ABI Prism 7900 Sequence Detector System (Applied Biosystems). Each sample was assayed in triplicate. The data were analyzed with SDS2.2 (ABI). The amount of each transcript was normalized with 18S and GAPDH levels.

### Measurement of autoantibodies

Patients' sera were tested for antinuclear antibodies by indirect immunofluorescence (IIF) using HEp-2 cells as antigen substrate and fluorescent goat anti-human IgG as a secondary antibody (Antibodies Inc., Davis, CA, USA). Anti-topo I antibodies were detected by passive immunodiffusion kits that employed calf thymus extracts as the antigen source (INOVA Diagnostics, San Diego, CA, USA), anti-RNA polymerase III antibodies were detected by ELISA using commercial kits (MBL, Nagoya, Japan). Anti-centromere antibodies were determined visually by their distinctive IIF patterns on HEp-2 cells. Anti-fibrillarin antibodies were detected by immunoprecipitation as described previously [16].

## Results

### Reduced catalytic function of topo I in SSc fibroblasts

**Figure 1:**
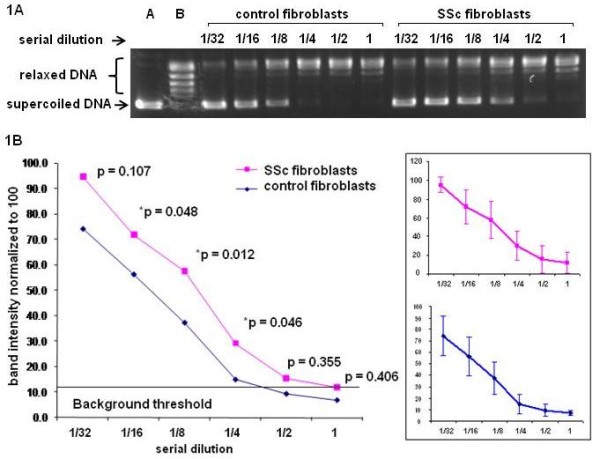
**Measurement of catalytic function of topo I in cultured fibroblasts**. A serial dilution of topo I in the nuclear extracts obtained from SSc and control fibroblasts was used to relax 0.25 μg supercoiled DNA. **A**. The supercoiled DNA band is gradually diminished following increased amounts of topo I in the nuclear extracts in the relaxing assays. The efficiency of SSc topo I in relaxing the supercoiled DNA appeared to be less than that of control topo I in each concentration of nuclear extracts. **B**. Comparison of 11 paired SSc and control fibroblasts for mean values of intensity of supercoiled DNA bands after relaxing assay with different concentrations of topo I in the nuclear extracts. Each *P-*value of comparison at different dilution points is listed in the figure. Comparison of average band intensity of remaining supercoiled DNA in each of six dilutions between all SSc and all control fibroblasts showed a significant *P-*value (*P *= 0.0041) (Student's *t*-test). A = standard supercoiled DNA band; B = standard relaxed DNA bands; the numbers (1/32, 1/16, 1/8, 1/4, 1/2 and 1) indicate serial dilutions of topo I in nuclear extracts used for relaxing supercoiled DNA. The error bars indicate standard deviation (SD).

After catalytic reactions with a serial dilution of topo I in the nuclear extracts, the supercoiled DNA band was gradually diminished following increased amounts of topo I in the nuclear extracts. Based on the intensity of supercoiled DNA bands that were correlated with the amounts of topo I in the nuclear extracts, the efficiency of SSc topo I in relaxing the supercoiled DNA appeared to be less than that of control topo I in each concentration of nuclear extracts (Figure 1A). Comparison of average band intensity of remaining supercoiled DNA in each of six dilutions between all SSc and all control fibroblasts showed a significant *P *value (*P *= 0.0041) (Student's *t*-test) (Figure 1B).

### Altered localization of topo I in SSc fibroblasts

**Figure 2:**
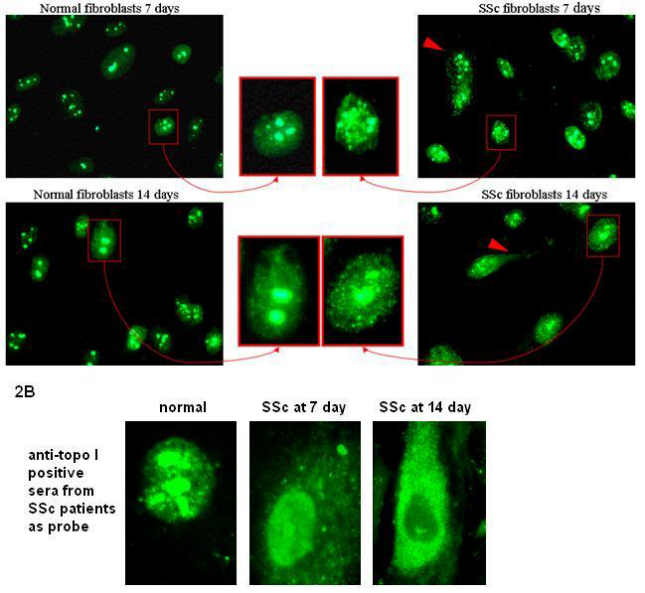
**Comparison of topo I staining in cultured fibroblasts of normal controls and SSc patients**. **A**. Topo I immunostaining with anti-topo I monoclonal antibodies showed multiple speckles in the nucleoplasm of SSc fibroblasts, which is differentiated from that in normal fibroblasts (relatively homogenous stain of topo I) at both 7 and 14 days of cultures. Some SSc fibroblasts show cytoplasmic staining of topo I protein (marked with red arrow heads). **B**. Topo I immunostaining with anti-topo I positive sera from SSc patients show the expected nuclear/nucleolar staining as well as cytoplasmic staining of SSc fibroblasts. At Day 14, the cytoplasmic staining appeared to increase relative to the nucleoplasm and nucleolar staining.

When anti-topo I monoclonal antibodies were used as probes, the majority of SSc fibroblasts from each patient showed strong nucleoplasmic staining (multiple speckles) compared to normal fibroblasts in which topo I staining was enriched in the nucleolus (Figure 2A). A few SSc fibroblasts (less than 1%) showed cytoplasmic (cytosolic) staining which was not observed in normal fibroblasts. However, there were more SSc fibroblasts (approximately 2%) showing cytoplasmic staining of topo I molecules when anti-topo I positive sera from SSc patients were used as probes (Figure 2B). The cytoplasmic staining of topo I appeared to be stronger at 14 or 18 days of culture compared to 7 days.

### Altered sumoylation of topo I in SSc fibroblasts

**Figure 3:**
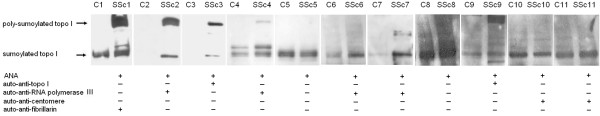
**Immunoprecipitated Western blots and autoantibody profiles for 11 SSc patients**. Each SSc patient (SSc1 to 11) has an age and sex matched normal control (C1 to 11) for comparison of sumoylated topo I expression with IP Western blots. Poly-sumoylated topo I appeared in SSc fibroblast strain number 1, 2, 3, 4, 7 and 9. Increased sumoylation of topo I also is observed in the case number 6 compared to its normal counterpart, but not in the case number 5, 8, 10 and 11. ANA, antinuclear antibodies.

Western blotting showed that the quantitative levels of topo I proteins were similar between SSc and normal control fibroblasts, while SUMO 1 levels were increased in SSc fibroblasts. To validate this finding, we examined sumoylated topo I in the nuclear proteins using IP Western blotting (Figure 3). Increased sumoylation of topo I (higher intensity of the bands and presence of poly-sumoylation of topo I) evaluated by IP Western blots was clearly observed in 7 of 11 SSc fibroblast strains (2 anti-topo I positive patients, 4 anti-RNA polymerase III positive patients and 1 anti-fibrillarin positive patient (Figure 3). Interestingly, four SSc fibroblast strains, including two each from patients with anti-centromere and with no detectable SSc specific autoantibodies, showed similar levels of sumoylation as their normal counterparts.

### Inhibition of SUMO1 in SSc fibroblasts increased catalytic function of topo I

**Figure 4:**
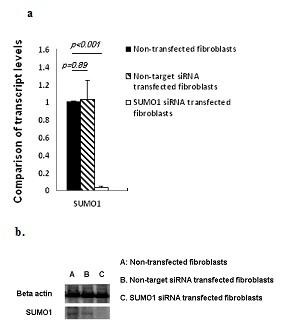
**Real-time RT-PCR and Western blots for SUMO1 with and without SUMO1 siRNA transfection in fibroblasts**. Three SSc fibroblast strains (two with anti-topo I and one with anti-RNA polymerase III positive serum) were transfected with SUMO1 siRNA. After 48-hour transfection, total RNAs were used for measuring SUMO1 transcript levels (Figure 4a), and the nuclear extracts were used for measuring SUMO1 protein (Figure 4b). Error bars indicate standard deviation.

**Figure 5:**
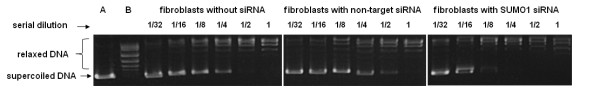
**Catalytic function of topo I in cultured SSc fibroblasts with and without SUMO1 siRNA transfection**. A serial dilution of the nuclear extract containing topo I obtained from SSc fibroblasts was used to relax 0.25 μg supercoiled DNA. In this figure, the supercoiled DNA band was completely transformed to relaxed DNA at dilutions of one half and one in the fibroblasts without siRNA transfection or non-target siRNA transfection. In contrast, this change was observed between the one-eighth and one-fourth dilutions in the fibroblasts with SUMO1 transfection, which indicates a higher efficiency of catalytic function of topo I after SUMO1 inhibition in the fibroblasts. According to the intensity of the bands of remaining supercoiled DNA in serial dilutions in the assays of three fibroblast strains, these changes are significant. The *P-*values are 0.045 and 0.027 at the one-fourth dilution for comparisons between SUMO1 siRNA vs. non-target siRNA, or vs. without siRNA transfected fibroblasts, respectively (Student's *t*-test). This is representative of three SSc fibroblast strains examined in SUMO1 siRNA studies. *A, supercoiled DNA; **B**, relaxed DNA.

Real-time quantitative RT-PCR showed that inhibition of SUMO1 with siRNA achieved a significant reduction of gene expression of SUMO1 (Figure 4). Compared to non-target siRNA transfected fibroblasts, SUMO1 siRNA transfected fibroblasts showed a 30.97-times reduction of SUMO1 expression (*P *< 0.001, T test) (Figure 4a). Western blots showed a concordant change of the SUMO1 protein (Figure 4b). Importantly, compared to either non-target siRNA transfected or non-siRNA transfected fibroblasts, catalytic function of topo I of sumo1 siRNA transfected SSc fibroblasts showed a marked improvement in all three test fibroblast strains (Figure 5). Measurements of the COL1A2 gene expression with quantitative RT-PCR and collagen type I protein expression with Western blots did not show significant changes after SUMO1 siRNA transfection in the fibroblasts.

### Sumoylation of recombinant topo I decreased its catalytic function

**Figure 6:**
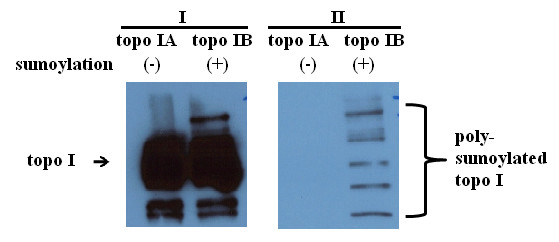
**Western blots show sumoylation of recombinant human topo I**. Recombinant human topo I protein was subjected to the sumoylation reaction and examined by Western blotting using anti-topo I (I) and anti-SUMO1 antibodies (II). Compared to topo I protein without sumoylation reaction (topo I **A**), topo I protein with sumoylation reaction (topo I **B**) showed poly-sumoylation of topo I (II). The assays showed similar results in triplicates.

**Figure 7:**
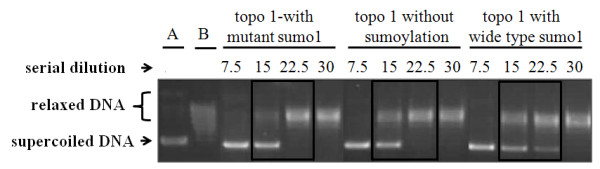
**Measurement of catalytic function of recombinant human topo I with and without sumoylation reaction**. Recombinant human topo I proteins were sumoylated with either mutant sumo1 or wild type sumo1 or negative control (without sumoylation), and then were examined for their catalytic function in a serial dilution. Sumoylation of topo I with wild type sumo1 showed a reduction of efficiency in catalytic function (supercoiled DNA disappeared at the dilution of topo I concentration of 30) compared to the topo I protein sumoylated with mutant sumo1 or negative control (supercoiled DNA disappeared at topo I concentration of 22.5). This is representative of three assays. *A, standard supercoiled DNA band; **B**, standard relaxed DNA bands.

Recombinant human topo I proteins were sumoylated with either wild type SUMO1 or mutant SUMO1 or negative control (without sumoylation) and then were examined with Western blot for sumoylated topo I and with topo I catalytic assays for topo I function. Poly-sumoylation of topo I was observed in the topo I proteins sumoylated with wild type SUMO1 (Figure 6). Sumoylation of topo I with wild type SUMO1 showed a reduction of efficiency in catalytic function compared to the topo I protein sumoylated with mutant sumo 1 or negative control (Figure 7). The assays were performed in triplicates, which showed similar results.

## Discussion

A novel finding of these studies is the observation that human SSc fibroblasts have a decreased catalytic function of topo I. Human topo I plays an important role in DNA metabolic processes, such as transcription and replication, in which it releases topological stress in DNA chains [9,10]. Topo I is generally localized in the nucleolus where a high level of transcription and replication of ribosomal DNA occurs. In response to inhibitory factors to topo I, such as camptothecin, UV irradiation and transcription inhibitors, topo I molecules were usually relocated from the nucleolus to the nucleoplasm due to mechanisms that are not clearly understood. [17-19]. Interestingly, SSc fibroblasts examined herein showed enhanced staining of topo I in the nucleoplasm, which suggests a relocation of topo I, and also supports a reduced function of topo I-associated DNA metabolic processes.

The cytoplasmic staining of topo I observed in some SSc fibroblasts was mainly detected by anti-topo I positive serum from SSc patients and was different from that found using anti-topo I monoclonal antibodies. Considering that the polyclonal human sera may contain mainly a variety of autoantibodies that have non-specific and antigen specific cross-reactions to cytoplasmic proteins is a possible explanation. With respect to possible cross-reactions, it is interesting that mitochondrial topo I has high amino acid homology to nuclear topo I. On the other hand, it is also possible that the cytoplasmic staining of topo I may represent ubiquitinated topo I molecules being processed by cytoplasmic proteasomes. It is worth noting that the topo I autoantigenic component, a 70 kD polypeptide, has been reported to be exported via ectocytosis in SSc fibroblasts [20], and anti-topo I autoantibodies of SSc patients have been shown to bind to SSc fibroblasts [21].

Sumoylation is an important post-translational modification. Previous studies have indicated that sumoylation of topo I facilitates translocation of topo I protein from the nucleolus to nucleoplasm [13,14]. Increased sumoylation of topo I in certain SSc fibroblasts observed herein supports a potential mechanism that may drive the movement of topo I from the nucleolar compartments to the nucleoplasm where a degradation process may occur in proteasomes. To further investigate the association between altered sumoylation and topo I function in SSc fibroblasts, we inhibited the SUMO1 expression with sequence specific SUMO1 siRNA. Interestingly, SUMO1 inhibition was associated with a favorable improvement of the catalytic function of fibroblast topo I, suggesting that decreased topo I function observed in SSc fibroblasts may be a result of increased sumoylation. This possibility was consistent with the follow-up studies of sumoylation of recombinant human topo I that showed a reduction of catalytic function. However, sumoylation may not fully explain the reduction of topo I function in all SSc fibroblasts, especially in those fibroblasts which did not show the changes of sumoylation of topo I. These fibroblasts include two each from patients with ACA and with non-SSc specific ANAs. In contrast, the fibroblasts from all seven patients with either anti-topo I ARA or anti-fibrillarin showed hyper-sumoylation of topo I. All these three autoantibodies target primary nucleolar proteins. It is worth noting that the presence of any one of these autoantibodies in SSc patients is associated with the diffuse form of SSc and internal organ fibrosis [8], while the anti-centromere positive patients usually have a limited form of SSc with favorable clinical outcomes [8]. Indeed, all SSc patients examined here with hypersumoylation of topo I presented as the diffuse form of SSc, except one, who was positive to ARA, but also clinically had lupus-like disease and anti-ribonucleoprotein (RNP) autoantibodies. All four SSc patients with unchanged sumoylation of topo I presented as the limited form of SSc at the time of skin biopsies. Therefore, sumoylaton of topo I in SSc fibroblasts appeared to be correlated with the status of skin fibrosis, which in some SSc patients changes over time. Recent studies of SSc genetics have indicated that different genetic susceptibility markers may determine the types of autoantibodies presenting in SSc patients [22,23]. The characteristic patterns and specific genetic associations of SSc autoantibodies suggest that distinctive mechanisms contribute to different autoantibody-associated SSc subsets.

Topo I is an essential functional component of human cells. Previous reports indicated that knock out of the topo I gene was associated with death at an early stage of embryogenesis [24,25]. Inactivation of the topo I gene *in vitro *was found to induce genomic instability with chromosomal aberrations [26]. Inhibition of topo I function through camptothecin or topotecan (a camptothecin derivative) in human HEp-2 cells altered nuclear structure and function and targeted topo I for proteasomal degradation [27]. Although, we do not know whether sumoylation of topo I in SSc fibroblasts contributes to any changes of specific antigen binding or autoantibody presentation in SSc patients, decreased catalytic function of topo I may alter the nuclear structure and function of the fibroblasts, which may influence other nuclear proteins including RNA pol III and fibrillarin. Of potential significance to our study, topotecan used therapeutically for cancer has been reported to induce SSc-like disease [28]. Whether decreased catalytic function of topo I in SSc fibroblasts examined herein may result in any consequences associated with pathological changes in SSc is worthy of further investigations.

## Conclusions

In summary, our studies of topo I in SSc fibroblasts indicate that topo I is functionally altered and is relocated to the nucleoplasm. In some fibroblasts, especially those obtained from skin biopsies of SSc patients who were positive for anti-topo I, anti-RNA polymerase III and anti-fibrillarin autoantibodies, these alterations were associated with increased sumoylation of topo I. In contrast, the fibroblasts of anti-centromere positive patients showed unchanged sumoylation of topo I. Inhibition of SUMO1 gene improved catalytic function of topo I in SSc fibroblasts. These observations may provide important insights into the nature of SSc fibroblasts that may contribute to pathological processes, induction of an autoimmune response to topo I, and/or disease development in SSc.

## Abbreviations

ANA: anti-nuclear antibodies; COL1A2: collagen type 1A2; DAPI: 4',6-diamidino-2-phenylindole; DMEM: Dulbecco's Modified Essential Media; ECL: Enhanced Chemiluminescence; ECM: extracellular matrix; FCS: fetal calf serum; GFP: green fluorescent protein; IIF: indirect immunofluorescence; IP: immunoprecipitation; RNP: ribonucleoprotein; SSc: systemic sclerosis; SUMO1: small ubiquitin-like modifier 1; Topo I: DNA topoisomerase I.

## Competing interests

The authors declare that they have no competing interests.

## Authors' contributions

ZX carried out research design, experiments and manuscript writing. WL conducted molecular studies and cell cultures. TF and AS conducted skin biopsies and helped with manuscript preparation. FM conducted autoantibody tests and manuscript preparation. GX carried out molecular studies and cell cultures. SR enrolled patients and did skin biopsies. XT conducted molecular studies. LS did skin biopsies for controls. AF carried out research design and manuscript preparation. All authors read and approved the final version of the manuscript.

## References

[B1] Tamby MC Chanseaud Y Guillevin L Mouthon L New insights into the pathogenesis of systemic sclerosis Autoimmun Rev 2003 2 152 157 10.1016/S1568-9972(03)00004-1 12848956

[B2] Steen VD The many faces of scleroderma Rheum Dis Clin N Am 2008 34 1 15 10.1016/j.rdc.2007.12.001 18329529

[B3] Claman HN Giorno RC Seibold JR Endothelial and fibroblastic activation in scleroderma. The myth of the "uninvolved skin" Arthritis Rheum 1991 34 1495 1501 1720957 10.1002/art.1780341204

[B4] Maul GG French BT van Venrooij WJ Jimenez SA Topoisomerase I identified by scleroderma 70 antisera: enrichment of topoisomerase I at the centromere in mouse mitotic cell before anaphase Proc Natl Acad Sci USA 1986 83 5145 5149 10.1073/pnas.83.14.5145 3014535 PMC323907

[B5] Diot E Giraudeau B Diot P Degenne D Ritz L Guilmot JL Lemarie E Is anti-topoisomerase I a serum marker of pulmonary involvement in systemic sclerosis? Chest 1999 116 715 720 10.1378/chest.116.3.715 10492277

[B6] Jarzabek-Chorzelska M Blaszczyk M Jablonska S Chorzelski T Kumar V Beutner EH Scl 70 antibody--a specific marker of systemic sclerosis Br J Dermatol 1986 115 393 401 10.1111/j.1365-2133.1986.tb06233.x 3535866

[B7] Kuwana M Kaburaki J Mimori T Kawakami Y Tojo T Longitudinal analysis of autoantibody response to topoisomerase I in systemic sclerosis Arthritis Rheum 2000 43 1074 1084 10.1002/1529-0131(200005)43:5<1074::AID-ANR18>3.0.CO;2-E 10817562

[B8] Bunn CC Black CM Systemic sclerosis: an autoantibody mosaic Clin Exp Immunol 1999 117 207 208 10.1046/j.1365-2249.1999.00990.x 10444248 PMC1905344

[B9] Leppard JB Champoux JJ Human DNA topoisomerase I: relaxation, roles, and damage control Chromosoma 2005 114 75 85 10.1007/s00412-005-0345-5 15830206

[B10] Wang JC Cellular roles of DNA topoisomerases: a molecular perspective Nat Rev Mol Cell Biol 2002 3 430 440 10.1038/nrm831 12042765

[B11] Li M Guo D Isales CM Eizirik DL Atkinson M She JX Wang CY SUMO wrestling with type 1 diabetes J Mol Med 2005 83 504 514 10.1007/s00109-005-0645-5 15806321

[B12] Meinecke I Cinski A Baier A Peters MA Dankbar B Wille A Drynda A Mendoza H Gay RE Hay RT Ink B Gay S Pap T Modification of nuclear PML protein by SUMO-1 regulates Fas-induced apoptosis in rheumatoid arthritis synovial fibroblasts Proc Natl Acad Sci USA 2007 104 5073 5078 10.1073/pnas.0608773104 17360386 PMC1829266

[B13] Rallabhandi P Hashimoto K Mo YY Beck WT Moitra PK D'Arpa P Sumoylation of topoisomerase I is involved in its partitioning between nucleoli and nucleoplasm and its clearing from nucleoli in response to camptothecin J Biol Chem 2002 277 40020 40026 10.1074/jbc.M200388200 12149243

[B14] Mo YY Yu Y Shen Z Beck WT Nucleolar delocalization of human topoisomerase I in response to topotecan correlates with sumoylation of the protein J Biol Chem 2002 277 2958 2964 10.1074/jbc.M108263200 11709553

[B15] Subcommittee for Scleroderma Criteria of the American Rheumatism Association Diagnostic and Therapeutic Criteria Committee Preliminary criteria for the classification of systemic sclerosis (scleroderma) Arthritis Rheum 1980 23 581 590 10.1002/art.1780230510 7378088

[B16] Arnett FC Reveille JD Goldstein R Pollard KM Leaird K Smith EA Leroy EC Fritzler MJ Autoantibodies to fibrillarin in systemic sclerosis (scleroderma). An immunogenetic, serologic, and clinical analysis Arthritis Rheum 1996 39 1151 1160 10.1002/art.1780390712 8670324

[B17] Buckwalter CA Lin AH Tanizawa A Pommier YG Cheng YC Kaufmann SH RNA synthesis inhibitors alter the subnuclear distribution of DNA topoisomerase I Cancer Res 1996 56 1674 1681 8603419

[B18] Christensen MO Krokowski RM Barthelmes HU Hock R Boege F Mielke C Distinct effects of topoisomerase I and RNA polymerase I inhibitors suggest a dual mechanism of nucleolar/nucleoplasmic partitioning of topoisomerase I J Biol Chem 2004 279 21873 21882 10.1074/jbc.M400498200 15014084

[B19] Mao Y Mehl IR Muller MT Subnuclear distribution of topoisomerase I is linked to ongoing transcription and p53 status Proc Natl Acad Sci USA 2002 99 1235 1240 10.1073/pnas.022631899 11805286 PMC122173

[B20] Hsu TC Lee TL Tsay GJ Autoantigen components recognizable by scleroderma sera are exported via ectocytosis of fibroblasts Br J Rheumatol 1997 36 1038 1044 10.1093/rheumatology/36.10.1038 9374918

[B21] Henault J Tremblay M Clement I Raymond Y Senecal JL Direct binding of anti-DNA topoisomerase I autoantibodies to the cell surface of fibroblasts in patients with systemic sclerosis Arthritis Rheum 2004 50 3265 3274 10.1002/art.20515 15476238

[B22] Zhou X Lee JE Arnett FC Xiong M Park MY Yoo YK Shin ES Reveille JD Mayes MD Kim JH Song R Choi JY Park JA Lee YJ Lee EY Song YW Lee EB HLA-DPB1 and DPB2 are genetic loci for systemic sclerosis: a genome-wide association study in Koreans with replication in North Americans Arthritis Rheum 2009 60 3807 3814 10.1002/art.24982 19950302 PMC2829245

[B23] Arnett FC Gourh P Shete S Ahn CW Honey R Agarwal SK Tan FK McNearney T Fischbach M Fritzler MJ Mayes MD Reveille JD Major Histocompatibility Complex (MHC) class II alleles, haplotypes, and epitopes which confer susceptibility or protection in the fibrosing autoimmune disease systemic sclerosis: analyses in 1,300 Caucasian, African-American and Hispanic cases and 1,000 controls Ann Rheum Dis 2010 69 822 827 10.1136/ard.2009.111906 19596691 PMC2916702

[B24] Lee MP Brown SD Chen A Hsieh TS DNA topoisomerase I is essential in Drosophila melanogaster Proc Natl Acad Sci USA 1993 90 6656 6660 10.1073/pnas.90.14.6656 8393572 PMC46991

[B25] Morham SG Kluckman KD Voulomanos N Smithies O Targeted disruption of the mouse topoisomerase I gene by camptothecin selection Mol Cell Biol 1996 16 6804 6809 8943335 10.1128/mcb.16.12.6804 PMC231683

[B26] Miao ZH Player A Shankavaram U Wang YH Zimonjic DB Lorenzi PL Liao ZY Liu H Shimura T Zhang HL Meng LH Zhang YW Kawasaki ES Popescu NC Aladjem MI Goldstein DJ Weinstein JN Pommier Y Nonclassic functions of human topoisomerase I: genome-wide and pharmacologic analyses Cancer Res 2007 67 8752 8761 10.1158/0008-5472.CAN-06-4554 17875716

[B27] Chen M Dittmann A Kuhn A Ruzicka T von Mikecz A Recruitment of topoisomerase I (Scl-70) to nucleoplasmic proteasomes in response to xenobiotics suggests a role for altered antigen processing in scleroderma Arthritis Rheum 2005 52 877 884 10.1002/art.20962 15751092

[B28] Ene-Stroescu D Ellman MH Peterson CE Topotecan and the development of scleroderma or a scleroderma-like illness Arthritis Rheum 2002 46 844 845 10.1002/art.10098 11920426

